# Carbon Dioxide Levels as a Key Indicator for Managing SARS-CoV-2 Airborne Transmission Risks Across 10 Indoor Scenarios

**DOI:** 10.7759/cureus.74429

**Published:** 2024-11-25

**Authors:** Narumichi Iwamura, Kanako Tsutsumi, Takafumi Hamashoji, Yui Arita, Takashi Deguchi

**Affiliations:** 1 Department of Nephrology, Japanese Red Cross Yamaguchi Hospital, Yamaguchi, JPN

**Keywords:** airborne transmission, co2 monitoring, covid-19, indoor air quality, infection probability, mask efficacy, sars-cov-2, wells–riley model

## Abstract

Background

The outbreak of severe acute respiratory syndrome coronavirus 2 (SARS-CoV-2) in December 2019 has led to a global pandemic through contact, droplets, and aerosolized particles.

Aim

This study aimed to quantify the airborne transmission risk of SARS-CoV-2 in various indoor environments.

Methods

Using indoor carbon dioxide (CO_2_) levels, we estimated the probability of airborne transmission and the basic reproduction number (R_0_) across 10 hypothetical indoor scenarios, including a college classroom, restaurant, classical music concert, live event, city bus, crowded train, hospital room, home, shogi match, and business meeting, using an analysis based on the modified Wells-Riley model.

Results

The relationship between airborne transmission rates and indoor CO_2 _concentrations was visualized with and without the use of masks. Without masks, at an indoor CO_2_ concentration of 1,000 ppm, airborne transmission rates were high in a home (100%), business meeting (100%), and hospital room (95%); however, they were moderate in a restaurant (55%), at a shogi match (22%), and at a live concert (21%); and low in a college classroom (1.7%), on a city bus (1.3%), at a classical music concert (1.0%), and on a crowded train (0.25%). In contrast, R_0_ was high at a live event (42.3), in a restaurant (15.9), in a home (3.00), and in a hospital room (2.86), indicating a greater risk of cluster infections. An examination of reduced airborne infection risk through surgical mask use and improved ventilation across various scenarios revealed that mask-wearing was highly effective in hospital rooms, in restaurants, at shogi matches, and in live concerts. Ventilation was particularly useful in hospital rooms, in restaurants, and at shogi matches.

Discussion and conclusion

In all indoor scenarios, a positive linear relationship existed between airborne transmission risk and indoor CO_2_ levels. The risk varied markedly across scenarios and was influenced by factors such as mask use, ventilation quality, conversation, and exposure duration. This model indicates that the risk of SARS-CoV-2 airborne transmission can be easily predicted using a CO_2_ meter.

## Introduction

Severe acute respiratory syndrome coronavirus 2 (SARS-CoV-2), which emerged in Wuhan, Hubei Province, China, in December 2019 [1], is responsible for the global dissemination of the disease known as coronavirus disease 2019 (COVID-19). Presenting with initial clinical manifestations that closely resemble those associated with influenza or rhinoviral infections, COVID-19 often eludes early diagnosis. The latency period between SARS-CoV-2 exposure and symptomatic manifestation spans one to 14 days, typically converging to a median of approximately five days. COVID-19 transmissibility precedes the clinical presentation of symptoms and amplifies shortly thereafter, which, along with its potential for asymptomatic transmission in the initial postexposure phase, has been implicated in widespread community-acquired infections, thereby complicating containment strategies.

SARS-CoV-2, like other respiratory pathogens, is typically transmitted through contact, respiratory droplets, or aerosolized particles [2]. Direct contact transmission involves the immediate transfer of viral particles via the tactile engagement of an infected individual, whereas indirect transmission may occur through contaminated inanimate surfaces, termed fomites. The production of respiratory droplets laden with viral particles occurs during expiratory activities, such as coughing, sneezing, and speaking; such droplets can then facilitate transmission to a susceptible host via mucosal surfaces. Note that this mode of transmission predominantly requires close interpersonal distance to ensure the deposition of droplets over short trajectories. Airborne transmission delineated as the conveyance of infectious agents - referred to as droplet nuclei within aerosolized particles - differs from droplet transmission in that these particles exhibit reduced dimensions, which enable prolonged suspension in air and transport over extended distances. Note that the concentration and particulate dimension of aerosols are modulated by the nature of respiratory activities (e.g., breathing, vocalization, coughing, and sneezing), influencing the vector dynamics of infection.

In our previous research, we assessed the airborne transmission risk of the SARS-CoV-2 Omicron variant within indoor settings, using carbon dioxide (CO_2_) levels as a proxy indicator [3]. This investigation introduced a refined variant of the Wells-Riley equation, which is traditionally used to estimate the probability of infection from airborne pathogens, augmented by incorporating measurements of indoor CO_2_ concentrations. The study’s pivotal contribution lies in adapting and applying the modified Wells-Riley model to quantify the risk of airborne transmission of SARS-CoV-2 predicated on the detected CO_2_ levels within indoor air. The model’s efficacy was validated by scrutinizing three case scenarios within a hospital, each delineating varied scenarios and ventilation conditions. The outcomes corroborated the model’s capacity as a robust tool for gauging infection probabilities in real-world scenarios, with significant implications for public health and safety protocols.

The research delineated specific CO_2_ concentration thresholds that, if sustained, inhibit the basic reproduction number (R_0_) of the virus from surpassing one, thus curbing the propensity for viral propagation in the environments under study. It denoted that in outpatient settings, CO_2_ concentrations maintained <620 ppm without masks, <1,000 ppm with surgical masks, and <1,600 ppm with N95 masks substantially reduced the risk of airborne transmission. Similarly, inpatient settings have corresponding thresholds set at <540 ppm without masks, <770 ppm with surgical masks, and <8,200 ppm with N95 masks. The study emphasized the crucial role of CO_2_ monitoring as a supplementary indicator of adequate ventilation and the potential risk of airborne infection. It underscored the importance of effective ventilation systems and the consistent use of protective masks in reducing the transmission risk of SARS-CoV-2 in hospital environments. The use of CO_2_ levels as a surrogate for appraising airborne transmission risk offers a pragmatic and attainable modality for healthcare entities and other indoor venues to evaluate and refine their air quality management tactics during the persistent COVID-19 pandemic and in anticipation of potential future respiratory viral epidemics.

Therefore, this study aimed to gauge the airborne transmission rate of SARS-CoV-2 across various nonhospital environments based on the modified Wells-Riley model, using graphical representations in various scenarios, including a college classroom, restaurant, classical music concert, live concert, city bus, crowded train, hospital room, home, shogi match, and business meeting. These representations will facilitate the use of CO_2_ concentration meters in making straightforward predictions of SARS-CoV-2 airborne transmission rates and discerning situations with elevated transmission risks. Additionally, we aim to provide actionable insights for public health policies, including specific examples of high-risk settings and the effectiveness of preventive measures such as CO_2_ monitoring, mask usage, and ventilation improvements.

## Materials and methods

Sample and data

In this study, no data sample collection or statistical analysis was performed.

Measures

We did not perform any actual measurements of data, including CO_2_ concentrations.

Models and data analysis

In this study, we used a modified Wells-Riley model that used indoor CO_2_ concentrations to estimate the probability of airborne transmission and R_0_ in the following 10 scenarios: a college classroom, restaurant, classical music concert, live concert, city bus, crowded train, hospital room, home, shogi match, and business meeting. The parameters for each scenario are set as shown in Table 1. 

**Table 1:** Parameters in 10 indoor environment scenarios

Scenario	Exposure time (hour)	Number of individuals staying in the room	Conversation
College classroom	1.0	30	No
Restaurant	1.0	30	Yes
Classical music concert	2.0	200	No
Live concert	2.0	200	Yes
City bus	0.50	20	No
Crowded train	0.50	100	No
Hospital room	24	4	No
Home	24	4	Yes
Shogi match	1.0	2	No
Business meeting	1.0	2	Yes

In this study, we derived a modified Wells-Riley model using indoor CO_2_ concentrations to estimate the probability of airborne transmission, which is consistent with our previous research. Riley et al. (1978) developed the Wells-Riley equation to estimate the probability of airborne transmission of infectious agents indoors [4]:

$P_{I}=\frac{C'}{S}=1-e^{-\frac{Iqpt}{Q}}$ (1)

where

P_I_ = infection probability (−)

C’ = number of susceptible individuals that were infected (−)

S = number of susceptible individuals (−)

I = number of infectious individuals (−)

q = generation rate of infectious quanta (/hour)

p = pulmonary ventilation rate of a person (m^3^/hour)

t = exposure time (hour)

Q = rate of room ventilation with outdoor air (m^3^/hour)

The Wells-Riley model requires the assumption that the air within a room is well mixed, ensuring that aerosols are distributed uniformly, thus focusing on airborne rather than droplet or contact transmission. Furthermore, the model does not account for the activation state of infectious particles. With the onset of the SARS-CoV-2 pandemic, face mask usage became prevalent in the Japanese population. The presence or absence of a mask on both infectious and susceptible individuals, along with the mask type - such as surgical or N95 - plays a crucial role in determining the probability of infection. Recognizing this, Dai and Zhao (2020) [5] introduced a revised version of the Wells-Riley model, which incorporates these significant variables.

$P_{I}=\frac{C'}{S}=1-e^{-\frac{Iqpt}{Q}\left( 1-\eta_{I} \right)\left( 1-\eta_{S} \right)}$ (2)

where

η_I_ = exhalation filtration efficacy (−)

η_S_ = respiration filtration efficacy (−)

The Wells-Riley model frequently presents challenges in application because it requires data on the rate of room ventilation with outdoor air (Q) - a value traditionally deduced from the performance metrics of ventilation fans and air conditioning systems. However, this ventilation rate is often subject to variations caused by factors such as the opening of doors and windows and the velocity and direction of the external wind, complicating the ability to yield precise estimates and consequently limiting the model’s usage. With regard to these challenges, this study estimated room ventilation rates by considering indoor CO_2_ concentration and emission rates. The Seidel formula, provided herein, delineates the relationship between room ventilation rates and indoor CO_2_ concentrations [6]:

$C_{o}\cdot Q'\cdot dt+M\cdot dt-C\cdot Q'\cdot dt=V\cdot dC$

$C=C_{o}+\left( C_{S}-C_{o} \right)\cdot e^{-\frac{Q}{V}t}+\frac{M}{Q}\cdot \left( 1-e^{-\frac{Q}{V}t}\right)$ (3)

where

C = indoor CO_2_ concentration (ppm)

C_0_ = atmospheric CO_2_ concentration (ppm)

C_S_ = initiation value of the indoor CO_2_ concentration (ppm)

Q' = rate of room ventilation with outdoor air per person (m^3^/hour/person)

V = room volume (m^3^)

t = time (hour)

M = CO_2_ emission rate of a person (m^3^/hour/person)

If the CO_2 _emission rate of a person (M) is constant and sufficient time has passed, the indoor CO_2_ concentration (C) stabilizes. Therefore, the rate of room ventilation with outdoor air per person (Q') is as follows:

$C=C_{o}+\frac{M}{Q'}$

$Q'=\frac{M}{C-C_{o}}$ (4)

where

Q' = rate of room ventilation with outdoor air per person (m^3^/hour/person)

C = indoor CO_2_ concentration (ppm)

C_0_= atmospheric CO_2_ concentration (ppm)

M = CO_2_ emission rate of a person (m^3^/hour/person)

The rate of room ventilation with outdoor air (Q) is as follows:

$Q = nQ'$ (5)

where

Q = rate of room ventilation with outdoor air (m^3^/hour)

n = number of individuals staying in the room (person)

The rate of room ventilation with outdoor air (Q) can be estimated by substituting Equation (5) for Equation (4) as follows:

$Q=n\frac{M}{C-C_{o}}$ (6)

The modified Wells-Riley model with indoor CO_2_ (C) can be obtained by substituting Equation (2) for Equation (6) as follows:

$P_{I}=\frac{C'}{S}=1-e^{\left\{-\frac{C-C_{O}}{M}\cdot \frac{Iqpt}{n}\left( 1-\eta_{I} \right)\left( 1-\eta_{S} \right) \right\}}$ (7)

$R_{o}=S\cdot P_{I}$ (8)

where

P_I_ = infection probability (−)

C' = number of susceptible individuals that were infected (−)

S = number of susceptible individuals (−)

C = indoor CO_2_ concentration (ppm)

C_0_ = atmospheric CO_2_ concentration (ppm)

M = CO_2_ emission rate of a person (m^3^/hour/person)

I = number of infectious individuals (−)

q = generation rate of infectious quanta (/hour)

p = pulmonary ventilation rate of a person (m^3^/hour)

t = exposure time (hour)

n = number of individuals staying in the room (−)

η_I_ = exhalation filtration efficacy (−)

η_s_ = respiration filtration efficacy (−)

R_0 _= basic reproduction number

To determine an acceptable level of individual exposure risk from a public health perspective - where minimizing outbreaks is paramount - R_0_ was used. R_0_ is defined as the expected number of secondary infections caused by a typical infector in a wholly susceptible population, and to mitigate the virus’s spread, the target exposure risk level must be set to R_0_ < 1 [7].

The parameters of the modified Wells-Riley model, which incorporates indoor CO_2_ data, include indoor CO_2_ concentration (C), ambient CO_2_ concentration (C_0_), an individual’s CO_2_ emission rate (M), the number of infectious individuals (I), the generation rate of infectious quanta (q), an individual’s pulmonary ventilation rate (p), exposure duration (t), the number of individuals present in the space (n), the exhalation filtration efficiency (ηI), and the inhalation filtration efficiency (ηS), as detailed in Table 2. The benchmark for indoor CO_2_ concentration (C) was established at 1,000 ppm, which is a level generally recognized in Japan as the demarcation between satisfactory and unsatisfactory ventilation; meanwhile, the ambient CO_2_ concentration (C_0_) was determined to be 417.9 ppm based on an analysis by World Meteorological Organization reported on November 15, 2023 [8].

**Table 2:** Types of parameters and reference values for the modified Wells-Riley model using indoor CO2 concentration NDIR, non-dispersive infrared

Parameters	References
Atmospheric CO_2_ concentration	Recommend measurement with a CO_2_ monitor employing NDIR method
Indoor CO_2_ concentration	Recommend measurement with a CO_2_ monitor employing NDIR method
Exposure time (hour)	-
CO_2_ emission rate for a person (m^3^/hour)	0.011 at rest, 0.01795 for talking [7]
Generation rate of infectious quanta (/hour)	20 for respiration, 1,535 for talking [9,10]
Pulmonary ventilation rate for a person (m^3^/hour)	0.48 for adult male [11]
Number of infectious individuals	-
Number of individuals staying in the room	-
Exhalation filtration efficacy	0 for no mask, 0.5 for surgical mask, 0.9 for N95 mask [12]
Respiration filtration efficacy	0 for no mask, 0.5 for surgical mask, 0.9 for N95 mask [12]
Results	-
Infectious probability (%)	-
Basic reproduction number (R_0_)	R_0_ >1 suggests the spread of virus infection [6]

Tajima et al. observed that a male adult’s CO_2_ emission rate (M) fluctuated between 0.011 and 0.0840, varying with the intensity of physical activity: 0.011 at rest, 0.0129-0.0230 during sedentary work, 0.0230-0.0330 when walking slowly, 0.0330-0.0538 for light labor, 0.0538-0.0840 for moderate labor, and >0.0840 for heavy labor [9]. They recommended adjusting these values by 0.9 for females and 0.5 for children. Therefore, in this study, we set the CO_2_ emission rates for a male adult accordingly.

An infectious quantum is a theoretical unit of infectivity derived from epidemiological research. It symbolizes the accumulation of viral particles necessary to initiate an infection. Prentiss et al. (2022) previously calculated the rate of generation of infectious quanta (q) using the Wells-Riley model for six instances of super-spreading events during the initial phase of the COVID-19 pandemic [10]. They reported a range for the estimated infectious quanta due to speaking between 136 and 757 per hour, averaging 461 per hour. However, considering the timing of the outbreak events analyzed, early SARS-CoV-2 strains were the focus of the study. Dai and Zhao estimated the infectious quanta to be 14 to 48 per hour, noting the variability among different SARS-CoV-2 variants [11]. The q for three variants - Alpha, Delta, and Omicron - was determined using a reproductive number-based fitting method applied to the Wells-Riley equation. The q values were 89-165 per hour for the Alpha variant, 312-935 per hour for the Delta variant, and 725-2,345 per hour for the Omicron variant. Based on these findings, we selected an estimated q value for speaking-associated emission of 1,535 per hour for the Omicron variant, representing the median of the estimated range. Conversely, Wang et al. assessed the infection probability within various types of aircraft cabins, assigning a q value of 20 per hour for breathing-related transmission [12]. Consequently, this study adopted a q value of 20 per hour for pulmonary ventilation rates, generally quantified at 0.48 m^3^ per hour [13].

Sickbert-Bennett et al. examined the filtration efficacy of hospital-grade face masks, establishing that even sub-optimally fitted N95 masks demonstrate efficacy exceeding 90% [14]. In contrast, surgical masks, whether secured with ties or ear loops, exhibit a reduced filtration efficiency ranging from 37% to 69%, which reflects their thinner filters and looser fit. In the current analysis, the exhalation filtration efficiency (η_I_) and inhalation filtration efficiency (η_S_) were set at 0% for unmasked individuals, 50% for surgical masks, and 90% for N95 masks. We have compiled the parameters of the modified Wells-Riley model and their reference values in Table 2.

## Results

College classroom

**Figure 1:**
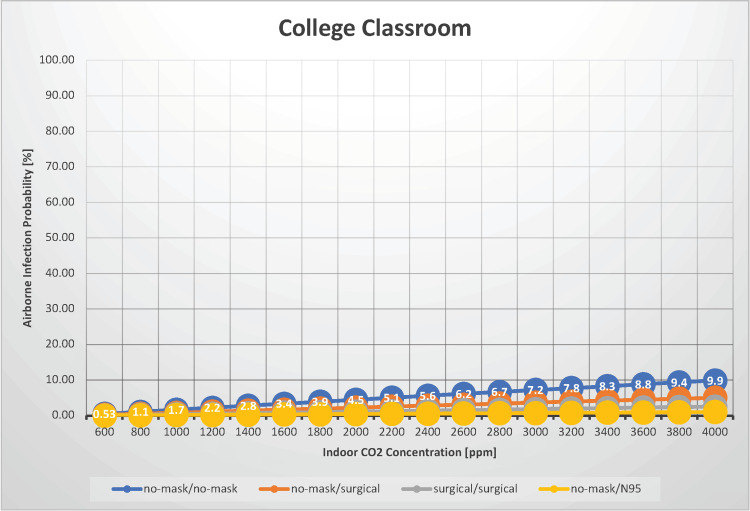
The relationship between indoor CO2 concentration and the probability of airborne transmission of SARS-CoV-2 in a college classroom

Infection with SARS-CoV-2 within educational institutions presents a more intriguing challenge. In this case, we estimated the airborne transmission rate of SARS-CoV-2 in a college classroom for indoor CO_2_ concentrations ranging from 600 to 4,000 ppm (Figure 1).

According to a report by Hou et al. on indoor air quality assessment in two primary schools, the average indoor CO_2_ concentration during class time with all doors and windows closed ranged from 1,285 to 1,659 ppm, whereas it averaged 1,153 ppm with doors and windows open. Moreover, the peak indoor CO_2 _concentration observed in this study was approximately 2,500 ppm [15]. A study measuring indoor CO_2_ levels under natural ventilation in Italian primary schools found that CO_2_ concentrations ranged from 782 to 4,064 ppm, with a median of 2,783 ppm [16]. Thus, even under the broad category of classrooms, CO_2_ concentrations can vary significantly depending on the room size, the state of windows and doors, the number of occupants, weather conditions, and wind speed, necessitating situation-specific assessments. The scenario was specifically named “college classroom” rather than simply “classroom” because the occupants were assumed to be adults. One key parameter in the SARS-CoV-2 infection prediction model is the pulmonary ventilation rate, which varies markedly with body weight, affecting the calculated infection probability. In this scenario, we used a conventionally accepted pulmonary ventilation rate of 0.48 m^3^/hour. In scenarios in which children constitute the majority of occupants, such as elementary or middle schools, the pulmonary ventilation rate is expected to be lower; thus, the infection probability and R_0_ are also expected to be lower. However, accurately predicting these values in populations with a wide range of body weights is challenging.

As mentioned, the average indoor CO_2_ concentration in a classroom setting is approximately 1,500 ppm, and even in poorly ventilated spaces, it typically does not exceed 3,000 ppm. For a scenario where 30 individuals participated in a 60-minute class, the airborne transmission probability was estimated to be between 0.53% and 9.9% when neither infectious nor susceptible individuals wore masks. When both parties wore surgical masks, the estimated airborne transmission probability ranged from 0.26% to 5.1%. Under normal ventilation conditions (with an indoor CO_2_ concentration of 1,000 ppm), the estimated airborne infection rate is 1.7% without and 0.4% with surgical masks. When attending a university lecture without a mask, individuals can focus with minimal concern for SARS-CoV-2 infection. For patients with immunodeficiency, wearing N95 masks can keep the airborne transmission probability under 1%, even in poorly ventilated spaces. However, these estimations assume complete silence during class; if the teacher is an infectious individual or if the class involves significant verbal communication or activities, such as singing, a higher airborne transmission rate should be anticipated. Furthermore, because the case scenarios in this study were based on a 60-minute class duration, regions with longer class durations should anticipate higher infection rates. For instance, the airborne transmission probability could be double the value presented in the graph for a 120-minute class duration. With an indoor CO_2_ concentration of 1,000 ppm, the estimated R_0_ is 0.153 without masks and 0.123 with surgical masks, indicating that SARS-CoV-2 outbreaks due to airborne transmission are unlikely in college classrooms.

Restaurant

**Figure 2:**
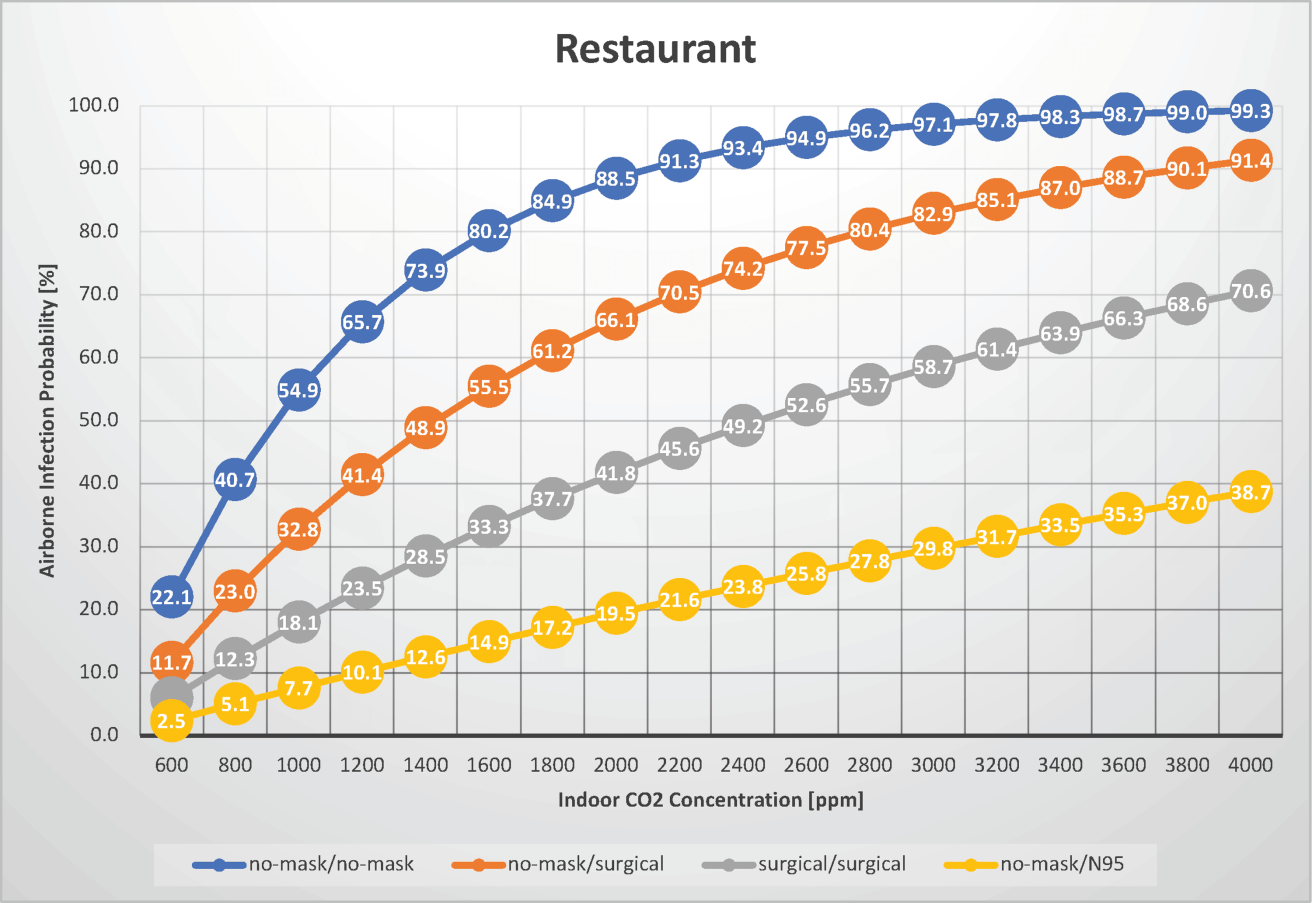
The relationship between indoor CO2 concentration and the probability of airborne transmission of SARS-CoV-2 in a restaurant

In this case, we estimated the airborne transmission rate of SARS-CoV-2 in a scenario where 30 individuals engage in conversation while dining for 60 minutes in a restaurant, with indoor CO_2_ concentrations ranging from 600 to 4,000 ppm (Figure 2).

The key difference from the college classroom scenario is the act of speaking, which is intrinsic to the restaurant setting. Restaurants enforcing a silent or no-talking policy during meals may find the college classroom scenario more applicable. Lee et al. investigated indoor air quality across four restaurant types in metropolitan Hong Kong, including a Korean barbecue restaurant, a Chinese hotpot restaurant, a Chinese dim sum restaurant, and a Western-style canteen. They found indoor CO_2_ concentrations ranging from 636 to 2,344 ppm [17].

The estimated airborne transmission probability without masks for both infectious and susceptible individuals ranged from 22% to 99%. When infectious and susceptible individuals wore surgical masks, the transmission probability ranged from 6.0% to 71%, indicating a moderate likelihood of transmission under such conditions. The probability of airborne transmission increases as the indoor CO_2_ concentration increases, underscoring the importance of maintaining good ventilation. Under normal ventilation conditions (with an indoor CO_2_ concentration of 1,000 ppm), the airborne infection risk is 55% without a mask, whereas wearing a surgical mask reduced the risk to 18%. However, wearing a mask continuously is challenging, as eating requires mask removal. Furthermore, partitions are not always installed between individuals; in such cases, droplet transmission must also be considered. Thus, restaurant settings are high-risk locations for SARS-CoV-2 infection. With an indoor CO_2_ concentration of 1,000 ppm, R_0_ is estimated to be 15.9 when neither party is masked and 5.24 even under surgical masks, suggesting that cluster outbreaks in restaurants could sustain transmission spread.

Classical music concert

**Figure 3:**
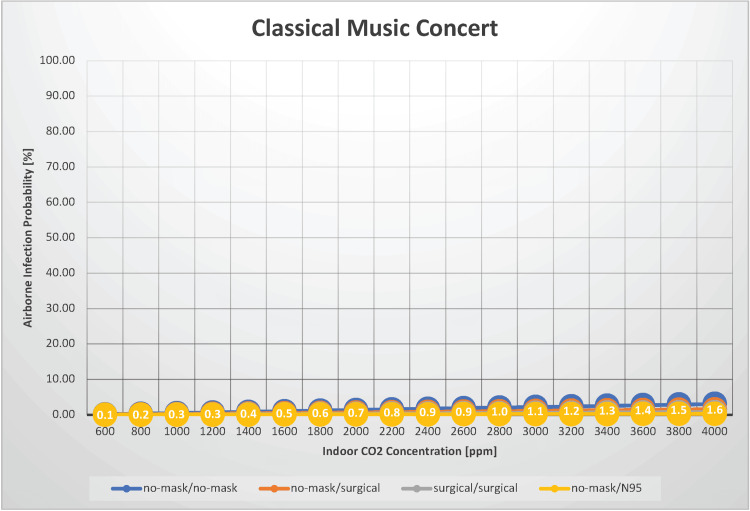
The relationship between indoor CO2 concentration and the probability of airborne transmission of SARS-CoV-2 in a classical music concert

Infection with SARS-CoV-2 during mass gathering events is a significant challenge. In this scenario, we estimated the airborne transmission probability of SARS-CoV-2 in an environment where 200 individuals are gathered for a two-hour period, such as at a classical music concert where the audience does not vocalize, with indoor CO_2_ concentrations ranging from 600 to 4,000 ppm (Figure 3).

Adzic et al. (2022) evaluated indoor air quality during mass gathering events. They reported that in one theater auditorium, with an attendance of 24% of the maximum capacity, the average CO_2_ concentration was 516 ppm, and the peak was 618 ppm. In another theater with 94% attendance, the average CO_2_ concentration reached 1,211 ppm, and the maximum was 1,617 ppm [18]. When neither infectious nor susceptible individuals wore masks, the airborne transmission probability was 0.16%-3.10%. When both parties wore surgical masks, the probability was 0.04%-0.78%. Under normal ventilation conditions (with an indoor CO_2_ concentration of 1,000 ppm), the estimated airborne infection risk is 0.51%, even when no one is wearing a mask. When attending a subdued concert, such as a classical music performance, individuals should not be concerned about the airborne transmission of SARS-CoV-2, even without a mask. However, if a nearby person frequently coughs or sneezes, the risk of airborne infection and droplet transmission will increase, substantially increasing the overall infection risk. With an indoor CO_2_ concentration of 1,000 ppm, the estimated R_0_ is 1.010 when no one is masked and 0.253 with surgical masks, indicating that an airborne outbreak of SARS-CoV-2 during a classical music concert is unlikely.

Live concert

**Figure 4:**
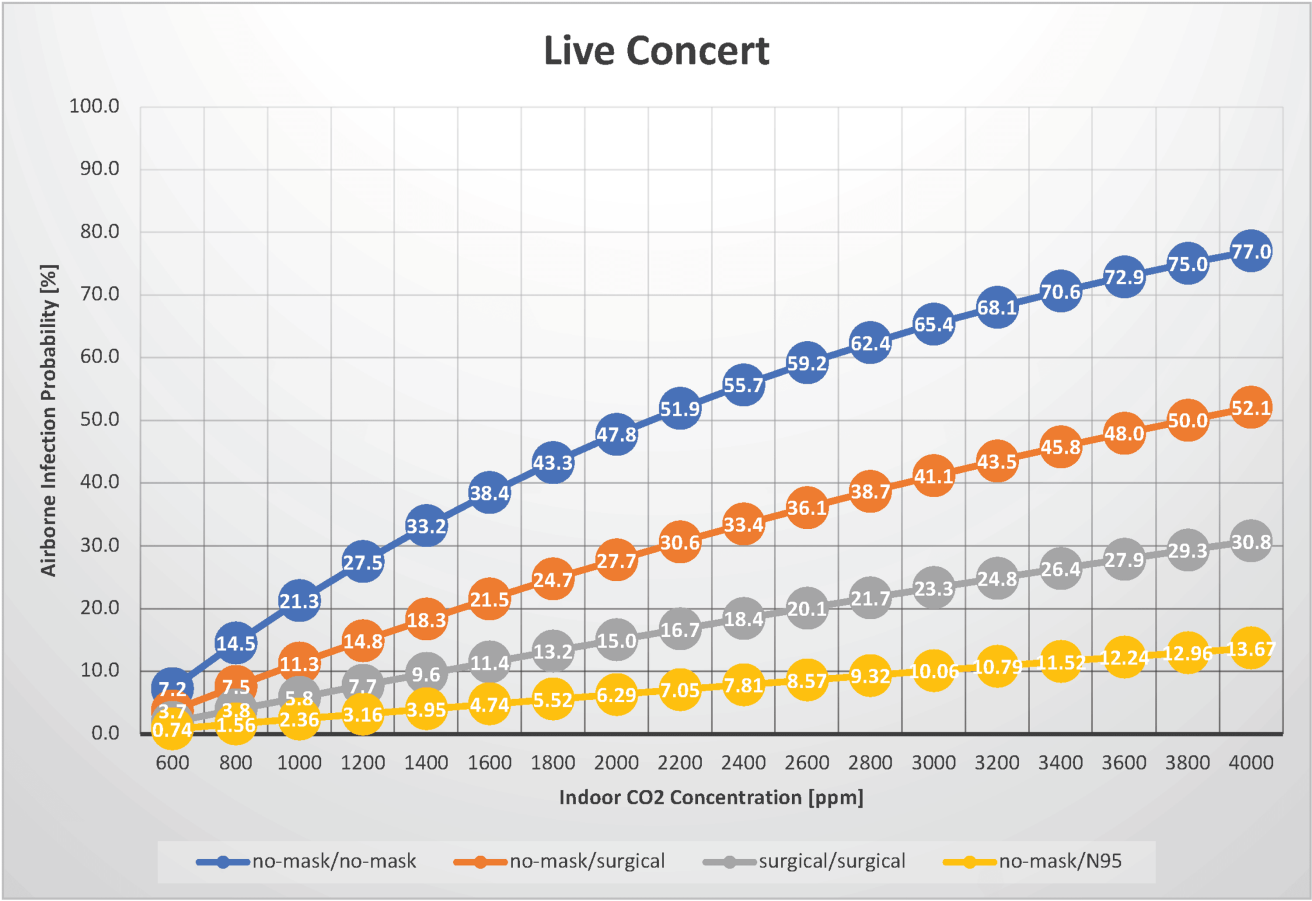
The relationship between indoor CO2 concentration and the probability of airborne transmission of SARS-CoV-2 in a live concert

In our study, we estimated the probability of airborne transmission of SARS-CoV-2 in a setting where 200 individuals gather for two hours, as might occur during a live show where the audience participates by vocalizing, with indoor CO_2_ concentrations between 600 and 4,000 ppm (Figure 4). 

This scenario also applies to indoor sports viewership. The primary difference from a classical music concert scenario is the presence of vocalization. If both infectious and susceptible individuals did not wear masks, the airborne transmission probability varied from 7.2% to 77%. This indicates a risk of up to 45 times higher than that associated with a classical music concert. The probability of airborne transmission increases as the indoor CO_2_ concentration increases, underscoring the importance of maintaining good ventilation. Moreover, audience participation in live concerts often involves louder speaking than normal conversation, which could elevate the generation rate of infectious quanta beyond established values. This has the potential to result in higher rates of airborne transmission than those initially estimated. Moreover, vocalization without mask usage, particularly in the context of a surgical mask not being worn, necessitates considering a significantly higher risk of droplet transmission, thereby increasing the estimated transmission probability. When both infectious and susceptible individuals wore surgical masks, the probability of airborne transmission ranged from 1.9% to 31%, suggesting a risk of up to 47.5 times that in a classical music concert scenario. Under normal ventilation conditions (with an indoor CO_2_ concentration of 1,000 ppm), the airborne infection risk without masks is moderate at 21%. Even when the infected person is unmasked, an uninfected person wearing a surgical mask can reduce the risk of airborne infection by approximately 11%, effectively halving the risk. With an indoor CO_2_ concentration of 1,000 ppm, R_0_ is estimated to be 42.3 when no masks are worn and 5.79 even when surgical masks are used, suggesting a high potential for cluster transmission in these scenarios. In summary, although the risk of individual airborne infection at a participatory live concert is moderate, a large number of attendees means that even a single infected person could trigger the largest cluster outbreak, making it a highly risky scenario.

City bus

**Figure 5:**
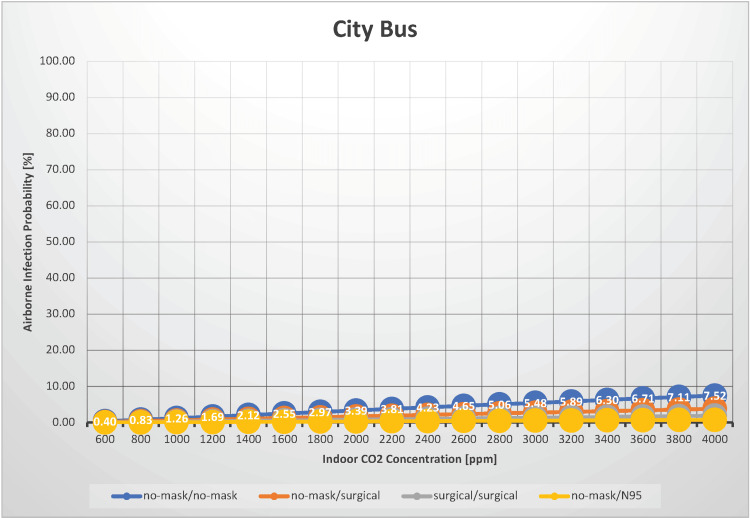
The relationship between indoor CO2 concentration and the probability of airborne transmission of SARS-CoV-2 in a city bus

Transmission of SARS-CoV-2 on public transportation is an intriguing issue. In our assessment, we estimated the probability of airborne transmission of SARS-CoV-2 in a scenario where 20 individuals were on a bus for 30 minutes, with indoor CO_2_ concentrations ranging from 600 to 4,000 ppm (Figure 5).

Moreno et al. measured CO_2_ concentrations in public transportation, reporting a median CO_2_ concentration inside buses of 674 ppm. [19]. When neither infectious nor susceptible individuals wore masks, the airborne transmission probability ranged from 0.40% to 7.52%. When both infectious and susceptible individuals wore surgical masks, the probability of transmission ranged from 0.10% to 1.93%. Under normal ventilation conditions (with an indoor CO_2_ concentration of 1,000 ppm), the probability of airborne transmission is relatively low at approximately 1.3%, even without a mask. Therefore, when riding a quiet bus where no one is talking, individuals can ride with peace of mind and little concern for SARS-CoV-2 infection, even when unmasked. However, this does not apply when an infected person is talking, coughing, or sneezing because the risk of airborne transmission and droplet infection increases substantially. In such cases, wearing a mask and maintaining a safe distance is advisable. Given an indoor CO_2_ concentration of 1,000 ppm, the estimated R_0 _is 0.0753 without and 0.0602 with surgical masks, indicating that the likelihood of a SARS-CoV-2 outbreak caused by airborne transmission on a city bus is extremely low.

Crowded train

**Figure 6:**
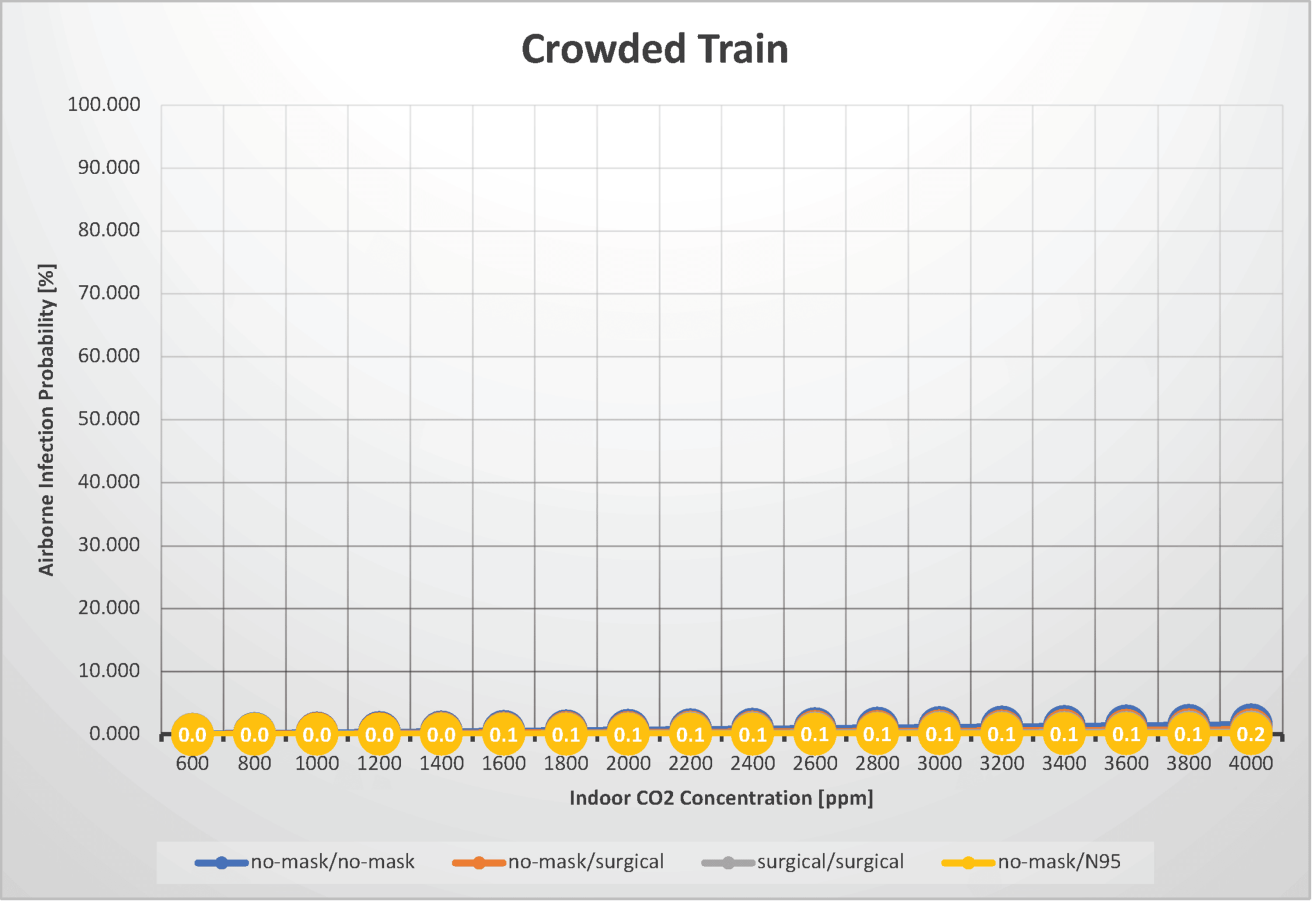
The relationship between indoor CO2 concentration and the probability of airborne transmission of SARS-CoV-2 in a crowded train

In this analysis, we estimated the airborne transmission probability of SARS-CoV-2 for a situation in which 100 individuals ride a crowded train for 30 minutes with indoor CO_2_ concentrations between 600 and 4,000 ppm (Figure 6). 

This scenario differs from that of a city bus in terms of the number of occupants: a city bus holds 20 individuals, whereas a crowded train setting holds 100 individuals. Shinohara et al. measured CO_2_ concentrations inside subway cars under simulated crowded operating conditions. Under conditions simulating 150% occupancy on an express service nonstop at intermediate stations, the CO_2_ concentration reached approximately 3,200 ppm with windows closed and approximately 2,700 ppm with windows slightly opened (10 cm × 2 locations) [20]. When neither infectious nor susceptible individuals wore masks, the airborne transmission probability ranged from 0.079% to 1.6%. When both individuals wore surgical masks, the probability ranged from 0.020% to 0.39%. These figures suggest that the potential for airborne transmission under these conditions is quite low. However, note that this estimation does not account for droplet transmission, and if the infectious person were to talk, cough, or sneeze, the risk of airborne transmission could significantly increase. In summary, when commuting on a crowded but quiet train, the airborne transmission risk is minimal, even without wearing a mask. However, if an unmasked, infected person is talking, coughing, or sneezing, the situation changes; in such cases, maintaining a sufficient distance from the infected person is advisable. If neither the infected person nor the potential virus recipient is wearing a mask and ventilation is normal (i.e., an indoor CO_2_ concentration of 1,000 ppm, the estimated R_0_ is 0.25. Therefore, SARS-CoV-2 clustering is unlikely to occur on a crowded but quiet train.

Hospital room

**Figure 7:**
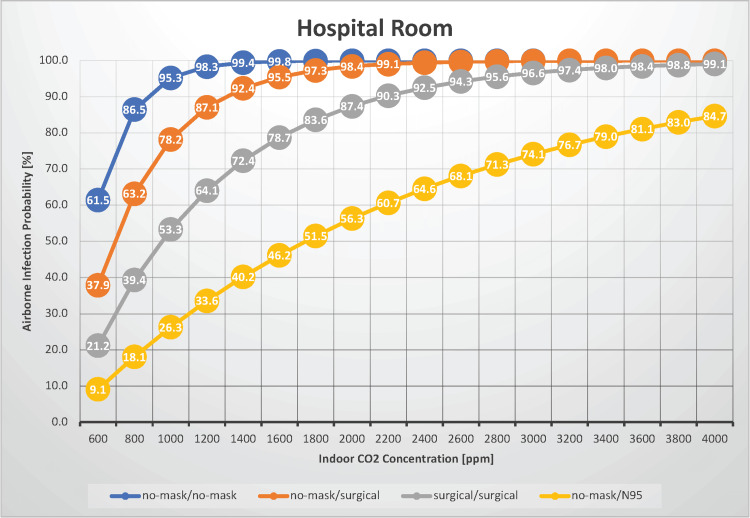
The relationship between indoor CO2 concentration and the probability of airborne transmission of SARS-CoV-2 in a hospital room

Nosocomial transmission of SARS-CoV-2 is a significant issue. In this scenario, we evaluated the probability of airborne transmission of SARS-CoV-2 in a situation where four individuals stayed in a hospital room for 24 hours, with indoor CO_2_ concentrations ranging from 600 to 4,000 ppm (Figure 7). 

We previously measured the indoor CO_2_ concentration in a four-person hospital room. The median CO_2_ concentration with three occupants was 1,187 ppm [3]. Without masks, the airborne transmission rate fluctuated between 62% and 100%. With both infectious and susceptible individuals wearing surgical masks, the transmission probability ranged from 21% to 99%. It becomes apparent that even without conversation between patients and with medical curtains dividing the beds, a 24-hour stay in a four-person room can result in a significantly high likelihood of airborne transmission. In practice, patients with respiratory diseases or cognitive disorders who cannot wear surgical masks all day or those with persistent coughing are common. Furthermore, the patient population often includes elderly or immunocompromised individuals, potentially raising the risk of airborne infection above the values discussed. Although patient spaces are typically segregated by medical curtains, which mitigates the risk of droplet transmission, the risk of contact transmission via shared facilities, such as toilets and sinks, must also be considered. Under normal ventilation conditions (with an indoor CO_2_ concentration of 1,000 ppm), the risk of airborne infection can reach 95% when no one is wearing a mask, whereas the risk remains high at 53% when surgical masks are worn. However, by ensuring adequate ventilation while all individuals are wearing surgical masks, the risk can be reduced to 21%, considered moderate risk. Therefore, if one patient in a shared hospital room develops COVID-19, it is highly likely that the other patients in the room are already infected, making spatial isolation and other precautions crucial. The estimated R_0_ value, given an indoor CO_2_ concentration of 1,000 ppm, is 2.86 when no masks are worn and 1.60 when surgical masks are worn, indicating a risk of nosocomial infection spread in such an environment. To prevent outbreaks within hospitals, healthcare workers should ensure sufficient ventilation in patient rooms and encourage patients who are able to wear surgical masks to do so as much as possible.

Home

**Figure 8:**
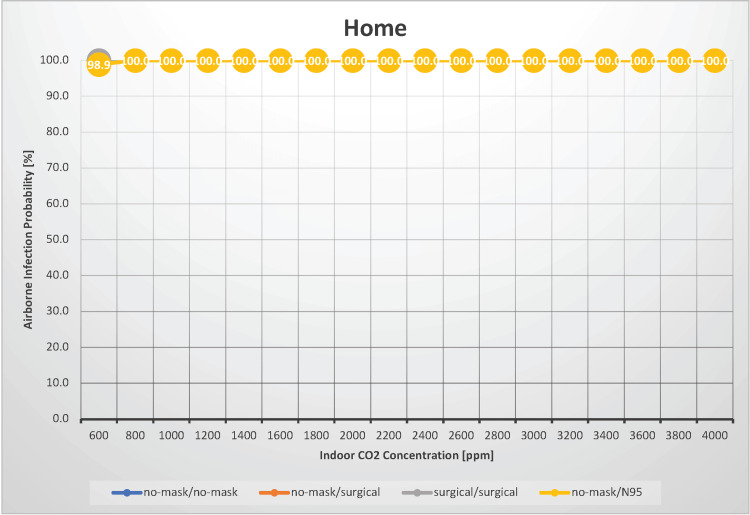
The relationship between indoor CO2 concentration and the probability of airborne transmission of SARS-CoV-2 in a home

Household transmission of SARS-CoV-2 frequently becomes a point of contention. In a scenario in which four individuals spent 24 hours at home, we estimated the probability of airborne transmission of SARS-CoV-2 with indoor CO_2_ concentrations ranging from 600 to 4,000 ppm (Figure 8). 

Schieweck et al. evaluated indoor air quality in smart homes and found that the median CO_2_ concentrations across various measuring points ranged from 587 to 1,360 ppm [21]. The primary difference from the hospital room scenario is the presence of vocalization, which occurs in the home setting. Regardless of whether the infectious or susceptible individuals wore masks, the risk of airborne transmission was 100%. Even with good ventilation (an indoor CO_2_ concentration of 600 ppm), the risk of airborne infection remains nearly 100%. In reality, considering the home environment, the risk of transmission likely increases because of the potential for droplet and contact transmission. In such a situation, if one family member is infected with SARS-CoV-2, it is highly probable that the infection will transmit to cohabitants. However, note that this estimation does not account for the immune status of the cohabitants; prior infection with SARS-CoV-2 or timing may not always lead to transmission. In this scenario, the estimated R_0_ at an indoor CO_2_ concentration of 1,000 ppm is 3.0, indicating a substantial risk of infection spread through household transmission.

Shogi match

**Figure 9:**
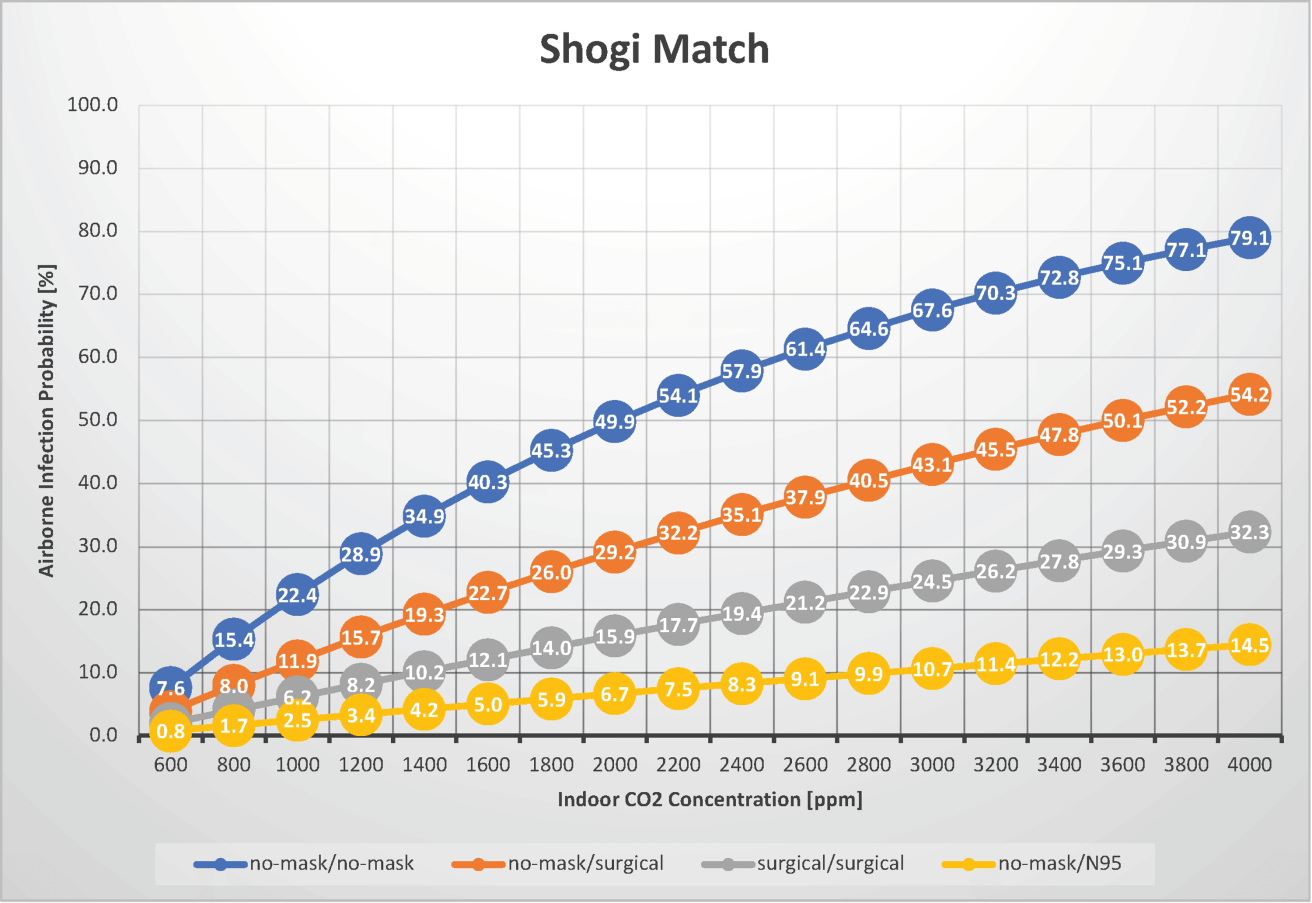
The relationship between indoor CO2 concentration and the probability of airborne transmission of SARS-CoV-2 in a shogi match

In a scenario in which two individuals spent 60 minutes together without conversing, such as during a shogi or chess match, we estimated the probability of airborne transmission of SARS-CoV-2 with indoor CO_2_ concentrations between 600 and 4,000 ppm (Figure 9). 

This situation could also apply to estimating the risk of airborne transmission in taxis, where there is minimal conversation between occupants. When neither the infectious nor susceptible individual wore masks, the airborne transmission probability ranged from 7.6% to 79%. The probability of airborne transmission increases as the indoor CO_2_ concentration increases, underscoring the importance of maintaining good ventilation. When both individuals wore surgical masks, the airborne transmission probability was 2.0%-32.0%. If both players in one-hour games of shogi or chess do not wear masks, the risk of airborne infection is 22% under normal ventilation conditions (an indoor CO_2_ concentration of 1,000 ppm). However, if ventilation is sufficient (an indoor CO_2_ concentration of 600 ppm), the risk of airborne infection is reduced to 7.6%. Conversely, if ventilation is poor (an indoor CO_2_ concentration of 2,000 ppm), the risk of airborne infection increases to 50%. If players prefer to focus on the game without wearing a mask, it is advisable to maintain an indoor CO_2_ concentration of <600 ppm. Moreover, if one player wears a surgical mask, the risk of airborne infection under normal ventilation conditions (an indoor CO_2_ concentration of 1,000 ppm) drops to approximately 6.2%, allowing both players to concentrate on the game with minimal concern for infection.

Business meeting

**Figure 10:**
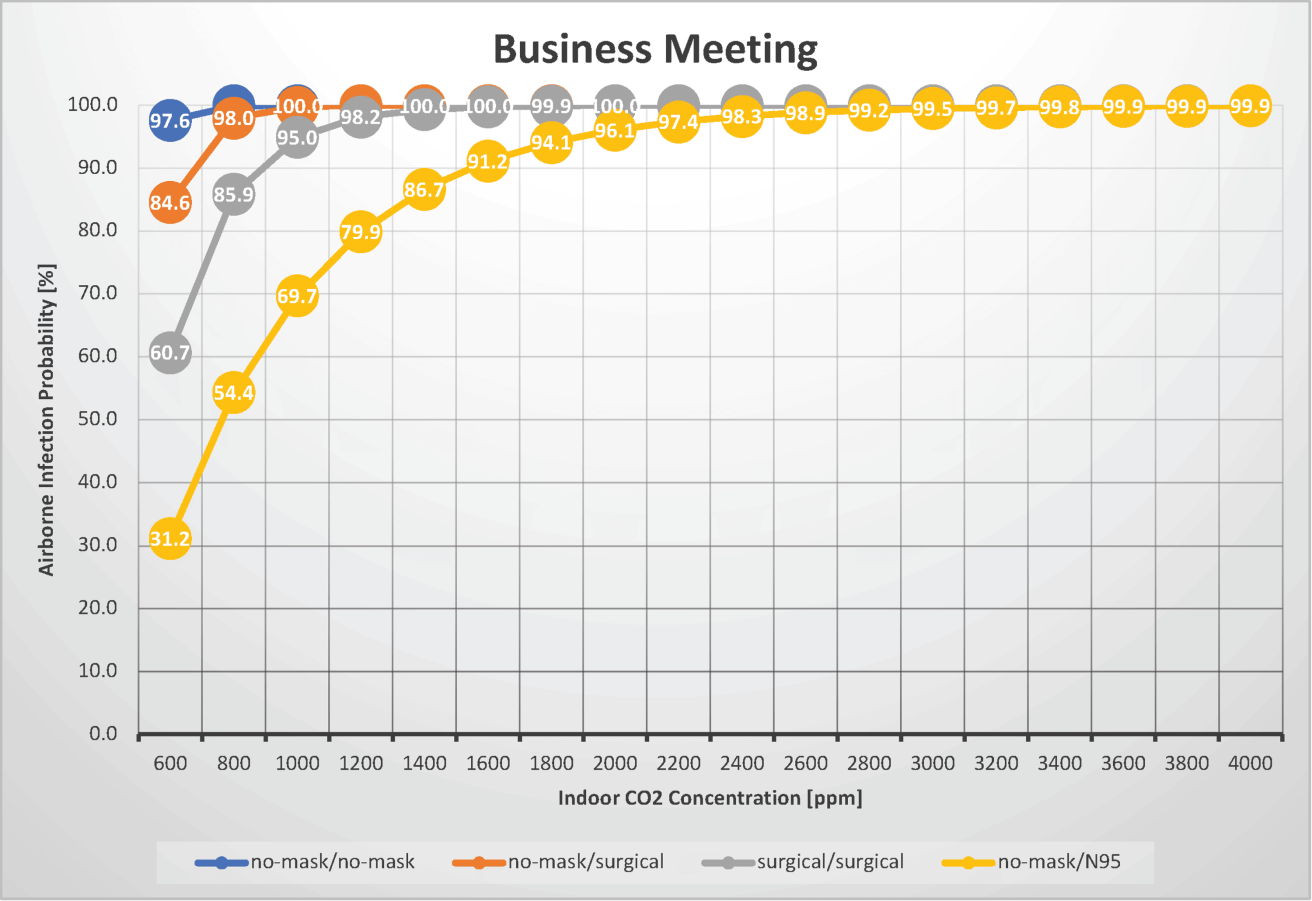
The relationship between indoor CO2 concentration and the probability of airborne transmission of SARS-CoV-2 in a business meeting

In a scenario where two individuals converse for 60 minutes, such as during a business meeting, we estimated the probability of airborne transmission of SARS-CoV-2 with indoor CO_2_ concentrations between 600 and 4,000 ppm (Figure 10). 

This scenario applies to other settings, including outpatient rooms at hospitals and situations where two acquaintances talk in a car or a tutor teaches a student at home. Our previously reported indoor CO_2_ concentrations for a hospital outpatient setting with five occupants, although not directly comparable, provide a reference with a median CO_2_ concentration of 1,116 ppm in a pediatric outpatient room and 549 ppm in a respiratory outpatient room. [3]. Similar conditions apply to outpatient hospital rooms. The difference from a shogi match is the presence of vocalization. If neither infectious nor susceptible individuals wore masks, the airborne transmission rate varied between 98% and 100%. When both parties wore surgical masks, the airborne transmission rate ranged from 61% to 100%. The transmission probability increases as the indoor CO_2_ concentration increases, reinforcing the need for good ventilation. Consider a scenario in which an individual is in a business meeting. If this individual and their counterpart wear surgical masks, the estimated infection probability is 61% with adequate ventilation (an indoor CO_2_ concentration of 600 ppm) but can reach 95% under normal ventilation conditions (an indoor CO_2_ concentration of 1,000 ppm). In such cases, maintaining sufficient ventilation is effective for reducing the risk of airborne transmission. However, the counterpart may not always be wearing a mask. If neither individual is wearing a mask, the airborne transmission risk remains high at 98%, even with good ventilation. In this case, wearing a surgical mask can reduce risk to 85%, whereas wearing an N95 mask can further reduce risk to 31%. However, as previously mentioned, this does not account for droplet or contact transmission and assumes that both individuals are separated by a partition. Without partitioning, the risk of airborne transmission is substantially increased.

## Discussion

The validity of the modified Wells-Riley model remains limited due to insufficient evidence. Although numerous cluster cases of airborne transmission of SARS-CoV-2 have been reported, very few cases include measurements of indoor CO_2_ concentrations. In a previous study, we retrospectively utilized the modified Wells-Riley model to estimate airborne infection rates and the R_0_ based on CO_2_ concentrations measured under identical conditions for three healthcare-related airborne transmission cases. For example, in a pediatric outpatient clinic where airborne transmission occurred, the actual airborne infection rate and the number of secondary cases were 75.0% and 3, respectively. On the other hand, the predicted airborne infection rate and R_0_ derived from the modified Wells-Riley model were 79.7% and 3.19, showing close agreement. Similarly, in a hospital ward with shared rooms, the actual airborne infection rate and secondary cases were 100% and 2, while the predicted values were 99.6% and 1.99, demonstrating near equivalence. Finally, in a respiratory outpatient clinic with no reported transmission, the actual airborne infection rate and number of secondary cases were 0%, whereas the model predicted 4.79% and 0.191, again showing close alignment [3]. These findings suggest that the modified Wells-Riley model provides a reasonably valid estimation of SARS-CoV-2 airborne infection rates and R_0_ under controlled conditions.

Additional real-world case studies further support the applicability of the modified Wells-Riley model for predicting SARS-CoV-2 airborne transmission under various scenarios. For instance, an airborne transmission case in a restaurant in China in January 2020 involved one index case potentially infecting nine secondary cases [22]. The airborne infection rate and R_0_ were calculated to be 11.5% and 9.0, respectively. Although CO_2_ concentrations were not directly measured, the predicted indoor CO_2_ concentration using the modified Wells-Riley model, based on a median exposure time of 68.5 minutes, was 702 ppm - a realistic value for relatively well-ventilated conditions. The relatively low infection rate of 11%, despite the restaurant's vulnerability to airborne transmission, may be partially attributed to effective indoor ventilation.

In another example, a choir rehearsal in the United States in March 2020 resulted in significant airborne transmission, with a single index case potentially infecting 52 secondary cases [23]. The airborne infection rate and R_0_ were estimated at 86.7% and 52, respectively. Although the CO_2_ concentration was not measured, the predicted value using the modified Wells-Riley model was 1,615 ppm, which is realistic for poorly ventilated conditions. The high transmission rate in this case can be attributed to prolonged, high-volume vocalizations characteristic of choir rehearsals, combined with insufficient ventilation, both of which likely contributed to the significant spread of SARS-CoV-2.

Similarly, a bus-related airborne transmission case in China in January 2020 involved an index case infecting nine secondary cases [24], with an airborne infection rate and R_0_ calculated at 15.0% and 9.0, respectively. While CO_2_ concentrations were not measured, the model initially estimated a predicted CO_2_ concentration of 4,950 ppm, a value that is unrealistically high. Upon adjusting the model to account for the likelihood of conversation by the index case - a reasonable assumption given the tourist bus setting - the predicted CO_2_ concentration dropped to 515 ppm, a realistic value indicative of good ventilation. These findings suggest that the observed transmission may have been due either to extremely poor ventilation or to the index case engaging in conversation, facilitating airborne transmission.

The influence of regional and seasonal variations in atmospheric CO_2_ concentrations on the modified Wells-Riley model's predictions was also evaluated. For example, CO_2_ concentrations measured at the Barrow, USA, monitoring station on August 1, 2023, and March 1, 2023, were 408.67 and 428.29 ppm, respectively. Similarly, at the Tae-ahn Peninsula in South Korea, CO_2_ concentrations were 414.32 ppm on August 1, 2023, and 432.49 ppm on March 1, 2023 [25]. Using these atmospheric CO_2_ values, the airborne transmission probabilities of SARS-CoV-2 during a live event were estimated. At Barrow, USA, under indoor CO_2_ conditions of 1,000 ppm, the transmission probability was 21.55% on August 1, 2023, and 20.91% on March 1, 2023. Similarly, at the Tae-ahn Peninsula, the probabilities were 21.36% and 20.78%, respectively. When compared to predictions based on the global atmospheric CO_2_ concentration of 417.9 ppm as of November 2023, the discrepancies ranged from -2.26% to 1.39%, suggesting that regional and seasonal differences in atmospheric CO_2_ concentrations have negligible impact on the model's predictions.

Comparison of the airborne transmission probability of SARS-CoV-2 among 10 scenarios

**Figure 11:**
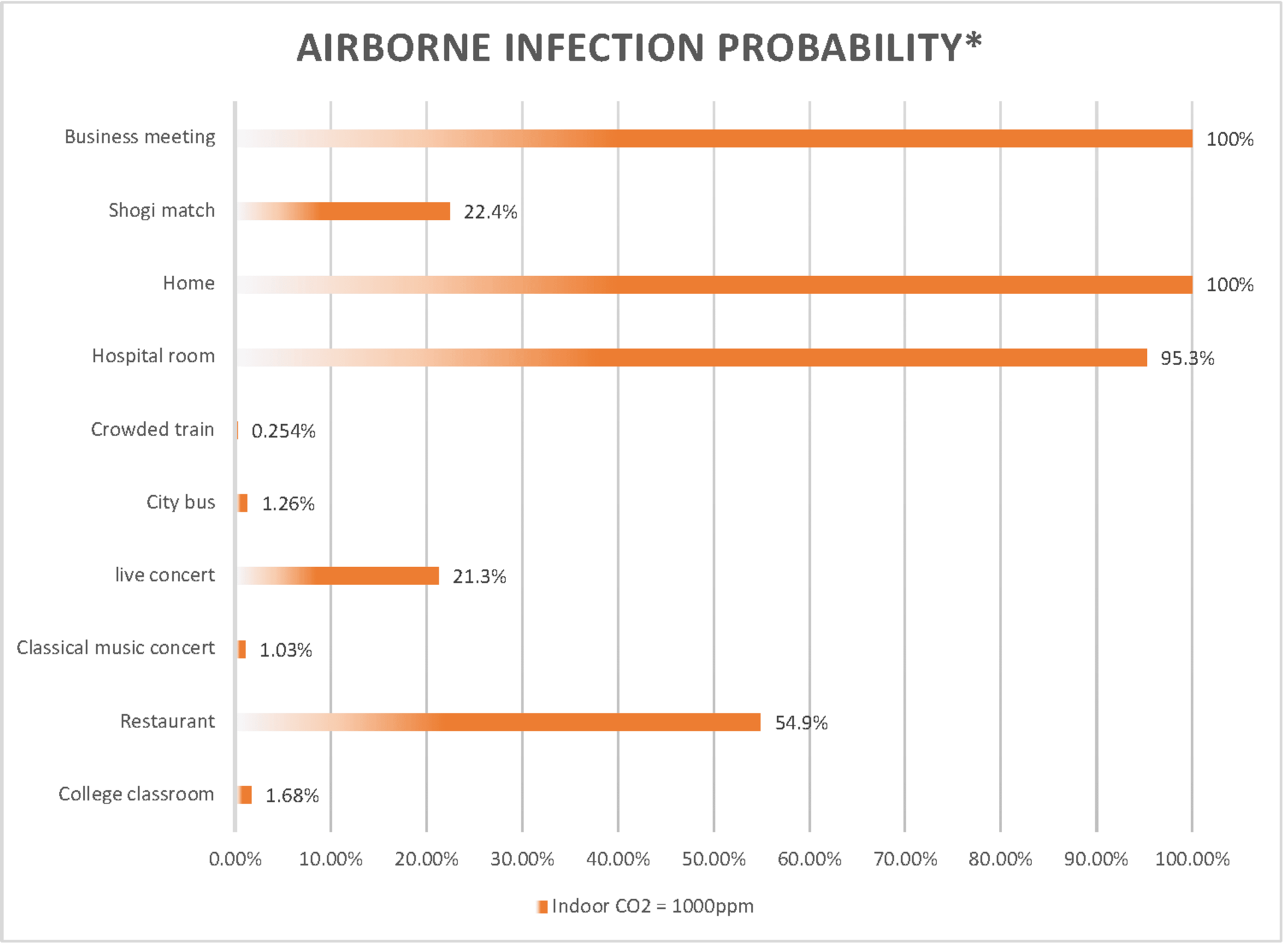
Comparison of the airborne transmission probability of SARS-CoV-2 under 1,000 ppm indoor CO2 concentration across 10 scenarios *The airborne transmission rates were estimated at an indoor CO_2_ concentration of 1,000 ppm across ten scenarios using a modified Wells-Riley model.

The indoor CO_2_ concentration is 1,000 ppm, and neither infected nor uninfected individuals are wearing any type of mask. Under these conditions, the probability of a single uninfected person contracting COVID-19 through airborne transmission is high in a business meeting (100%), at home (100%), in a hospital room (95%), and in a restaurant (55%). It is moderate at a live concert (21%) and in a shogi match (22%). It is low in a college classroom (1.7%), on a city bus (1.3%), at a classical music concert (1.0%), and on a crowded train (0.25%) (Figure 11).

The common characteristic of situations with a high risk of airborne transmission is that they involve talking or vocalization. Talking or vocalizing can result in 76.8 times more infectious quanta than breathing alone. (An infectious quantum is a hypothetical unit of infectivity derived from epidemiological studies, representing the assembly of viral particles necessary to establish infection.) Upon examining the characteristics of 19 reported cases of airborne SARS-CoV-2 transmission available as of December 2020 (Table 3) [22-24,26-38], it is evident that indoor environments involving conversation or similar actions (such as oxygen mask usage) tend to exhibit high transmission rates, even over relatively short periods. This pattern is observed in cases such as the karaoke room in China (Case 1) [26], the restaurant in China (Case 2) [22], and the choir rehearsal in the USA (Case 5) [23].

**Table 3:** Summary of airborne transmission cases of SARS-CoV-2 in 2020

Case no.	1	2	3	4	5	6	7	8	9	10	11	12	13	14	15	16	17	18
Reporter and reported year	Liu et al. 2020 [26]	Li et al. 2021 [22)	Hwang et al. 2020 [27]	Andres et al. 2022 [28]	Miller et al. 2021 [23]	Pung et al. 2020 [29]	Katelaris et al. 2021 [30]	Katelaris et al. 2021 [30]	Shen et al. 2020 [31]	Luo et al. 2020 [32]	Cheng et al. 2022 [24]	Cheng et al. 2022 [24]	Chen et al. 2020 [33]	Khanh et al. 2020 [34]	Hoehl et al. 2020 [35]	Choi et al. 2020 [36]	Speake et al. 2020 [37]	Bae et al. 2020 [38]
Date of infection cases	14-Jan-20	24-Jan-20	23-Aug-20	2-Dec-20	10-Mar-20	19-Jan-20	15-Jul-20	16-Jul-20	19-Jan-20	22-Jan-20	22-Jan-20	22-Jan-20	24-Jan-20	2-Mar-20	9-Mar-20	9-Mar-20	19-Mar-20	31-Mar-20
Country of infection cases	Wuhan, China	Guangzhou, China	Seoul, Korea	Terrassa, Spain	Washington, USA	Singapore	Sydney, Australia	Sydney, Australia	Zhejiang, China	Hunan, China	Hunan, China	Hunan, China	Singapore to Hangahou, China	London to Hanoi	Tel Aviv to Frankfurt	Boston to Hong Kong	Sydney to Perth	Milan to Seoul
Type of indoor space	Karaoke room	Restaurant	Apartment	Hospital	Choral rehearsal	Church	Church	Church	Bus	Bus	Bus	Bus	Flight	Flight	Flight	Flight	Flight	Flight
Exposure time	120 min	48-89 minutes	N/A	N/A	150 minutes	120 minutes	60 minutes	60 minutes	N/A	150 minutes	200 minutes	60 minutes	310 minutes	10 hours	280 minutes	15 hours	300 minutes	11 hours
Number of index cases	1	1	1	1?	1	2	1	1	1	1	1	1	2	1	7	2	18	6
Number of individuals staying in the same space	7	79	437	N/A	61	144	215	120	68	61	47	17	335	184	71	294?	64	310
Number of secondary cases	5	9	9	64	52	2	5	7	23	9	7	2	14	15	2	2	8	1
Conversation	Yes	Yes	No	Yes	Yes	N/A	Yes	Yes	N/A	N/A	N/A	N/A	No	No	No	N/A	No	No
Mask-wearing status of the infected individuals	No?	No?	No	No	No?	N/A	No	No	No	No	No	No	Yes	N/A	No	N/A	No	N95
Mask-wearing status of the susceptible individuals	No?	No?	No	N/A	No?	N/A	No	No	No	No	No	No	Yes	N/A	No	N/A	No	N95
Airborne infection probability	83.3%	11.5%	2.06%	N/A	86.7%	1.41%	2.40%	5.88%	34.3%	15.0%	15.2%	12.5%	4.20%	8.20%	3.13%	0.685%	14.3%	0.329%
Basic reproduction number (R_0_)	5.0	9.0	9.0	64?	52	1.0	5.0	7.0	23	9.0	7.0	2.0	7.0	15	0.29	1.0	0.44	0.17

Conversely, a high probability of airborne transmission was calculated in hospital rooms despite the absence of conversation, which can be attributed to the prolonged exposure time of 24 hours. As seen in the in-flight transmission cases from London to Hanoi (Case 14) [34] and from Sydney to Perth (Case 17) [37], prolonged exposure times can increase the likelihood of airborne transmission, even in the absence of conversation (Table 3). The moderate risk during a shogi match, where there is no conversation and only short-term exposure, is presumed to be because of relatively poor ventilation. In many cases, an indoor CO_2_ concentration of 1,000 ppm for two individuals indicates relatively poor ventilation. Therefore, choosing not to wear surgical masks in such situations poses a high risk. Conversely, the probabilities of infection on a crowded train, city bus, and in a college classroom are low. However, caution is warranted in interpreting these results. An indoor CO_2_ concentration of 1,000 ppm in scenarios with large numbers of individuals indoors - 100 on a bus, 20 on a train, and 200 in a college classroom - suggests quite good ventilation, indicating airborne transmission rates in very well-ventilated conditions. In reality, CO_2_ concentrations can increase to 1,300 ppm on a city bus, 3,200 ppm on a crowded train, and 1,600 ppm at a classical music concert, with the probabilities of airborne transmission estimated at 1.9%, 1.2%, and 1.0%, respectively. Although these rates are significantly lower than those in the other scenarios, they are not negligible. The risk of airborne transmission can increase substantially on a city bus, on a crowded train, or at a classical music concert if the infected person talks, coughs, or sneezes, and the risk of droplet infection also increases without the use of face shields or eye protection. Therefore, wearing surgical masks in these scenarios, as in other scenarios, is considered important.

**Figure 12:**
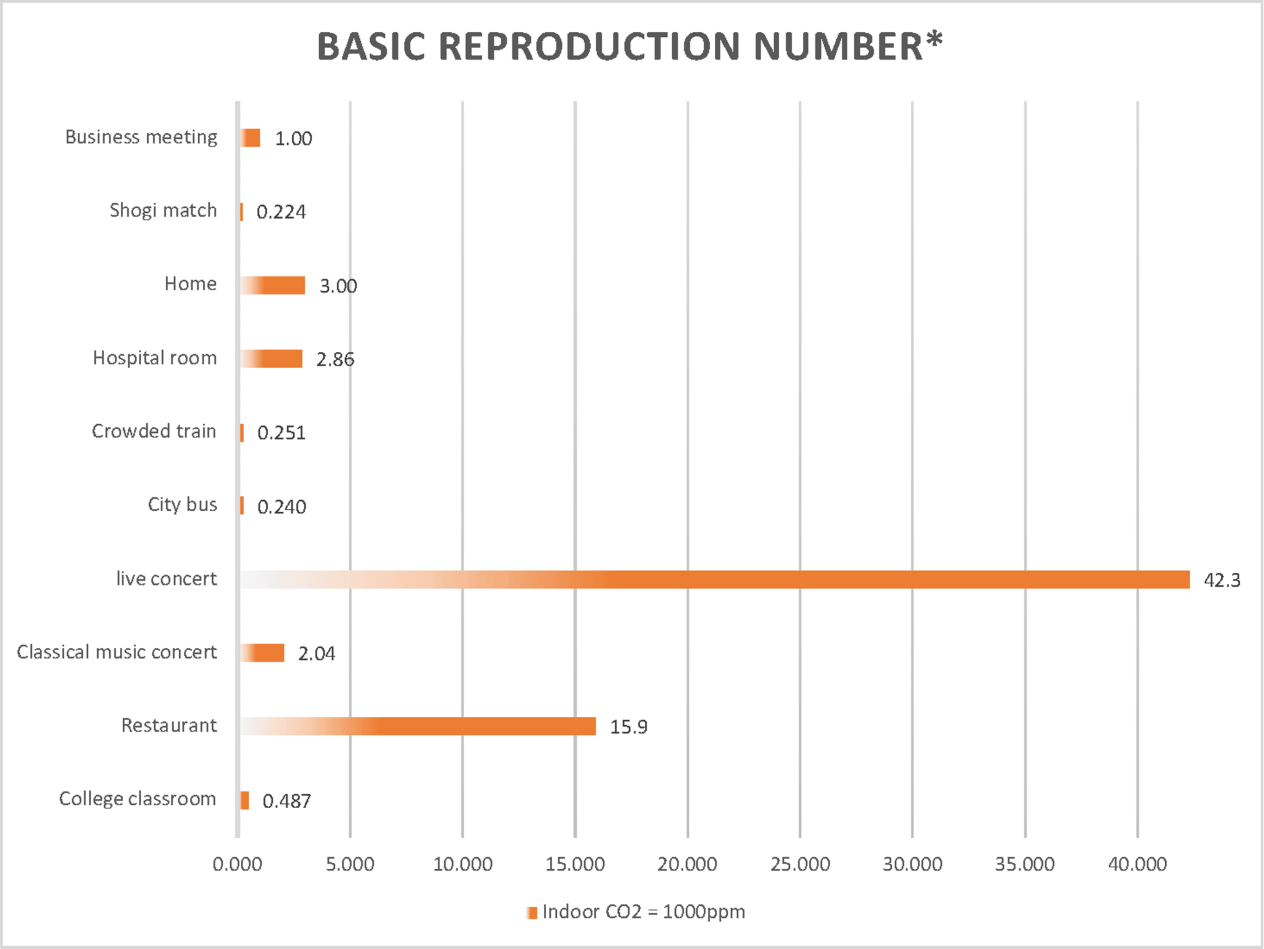
Comparison of the basic reproduction number of SARS-CoV-2 under 1,000 ppm indoor CO2 concentration across 10 scenarios *The basic reproduction numbers were estimated at an indoor CO_2_ concentration of 1,000 ppm across ten scenarios using a modified Wells-Riley model.

R_0_ estimates the average number of secondary infections produced by an infected individual. When this value exceeds one, it indicates that the infection is spreading. With an indoor CO_2_ concentration of 1,000 ppm and neither infected nor uninfected individuals wearing masks, the R_0_ is alarmingly high for a live concert (42.3) and restaurants (15.9), and it exceeds one in homes (3.00) and hospital rooms (2.86) (Figure 12). These scenarios highlight a very high risk of cluster infections, necessitating thorough precautionary measures. In the case of the choir rehearsal in the USA (Case 5) [23], it was reported that one primary infected individual led to 52 secondary infections over a 2.5-hour period. Similarly, in the restaurant setting in China (Case 2) [22], one primary infected individual resulted in nine secondary infections.

These situations, which are characterized by a high airborne transmission probability and several indoor occupants, are particularly prone to outbreaks. In contrast, scenarios such as a shogi match (0.224), crowded train (0.251), city bus (0.240), and college classroom (0.487), in which there are fewer opportunities for vocalization, may be less susceptible to widespread transmission.

**Figure 13:**
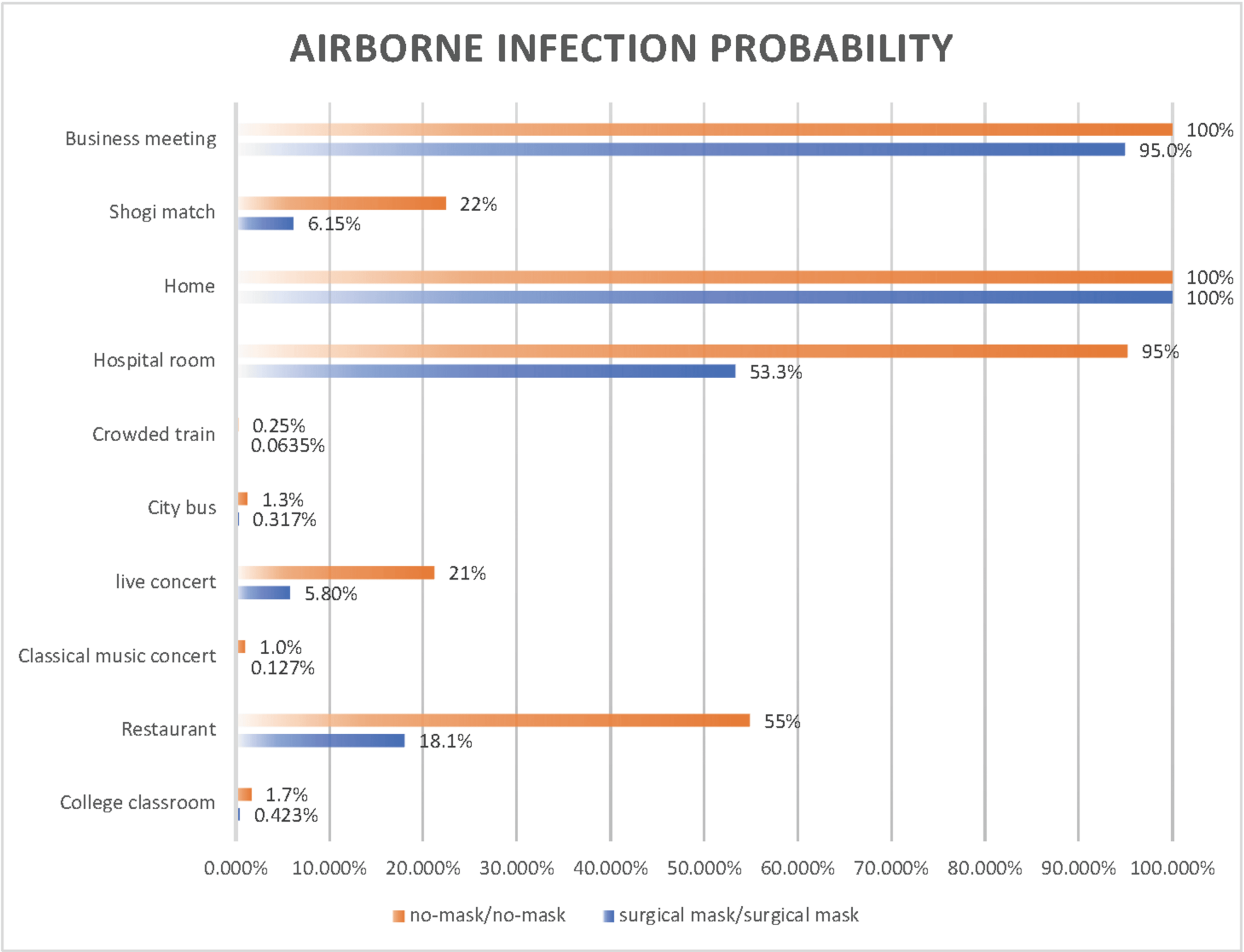
Comparison of the airborne transmission probability of SARS-CoV-2 with and without the use of surgical masks at an indoor CO2 concentration of 1,000 ppm

**Figure 14:**
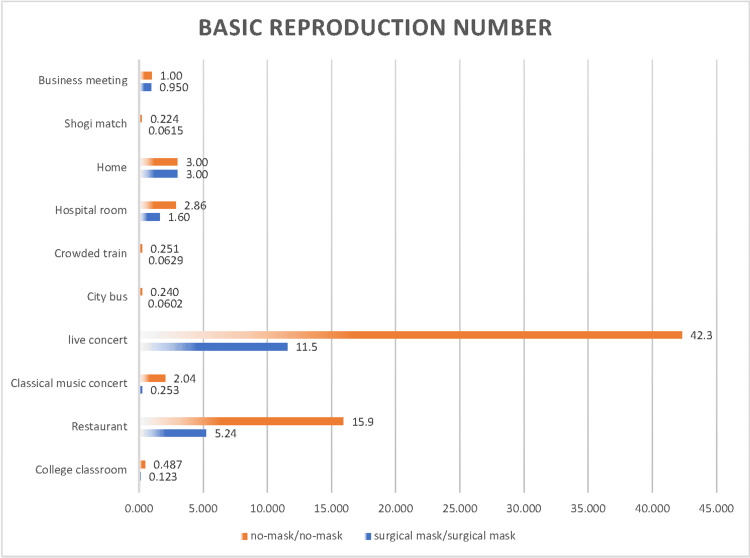
Comparison of the basic reproduction number of SARS-CoV-2 with and without the use of surgical masks at an indoor CO2 concentration of 1,000 ppm

The effectiveness of surgical masks in various scenarios was investigated (Figure 13 and Figure 14).

Estimates of the probability of airborne transmission were made for scenarios in which both noninfected and infected individuals did not wear any masks (indicated in orange) and for scenarios in which they wore surgical masks (indicated in blue), all at an indoor CO_2_ concentration of 1,000 ppm. The reduction in the probability of airborne transmission was significant in a hospital room (42%), restaurant (37%), shogi match (16%), and live concert (15%), indicating that the use of surgical masks is highly effective in these settings. In contrast, in other scenarios such as a college classroom (1.3%), classical music concert (0.90%), city bus (0.95%), crowded train (0.19%), home (0.0%), and business meeting (5.0%), no significant reduction in the risk of airborne transmission of SARS-CoV-2 due to mask-wearing was observed. However, this does not imply that wearing a surgical mask is useless in these scenarios. Particularly in high-risk scenarios, such as at home or in a business meeting, a combination of adequate ventilation and wearing surgical masks may effectively reduce infection risk.

**Figure 15:**
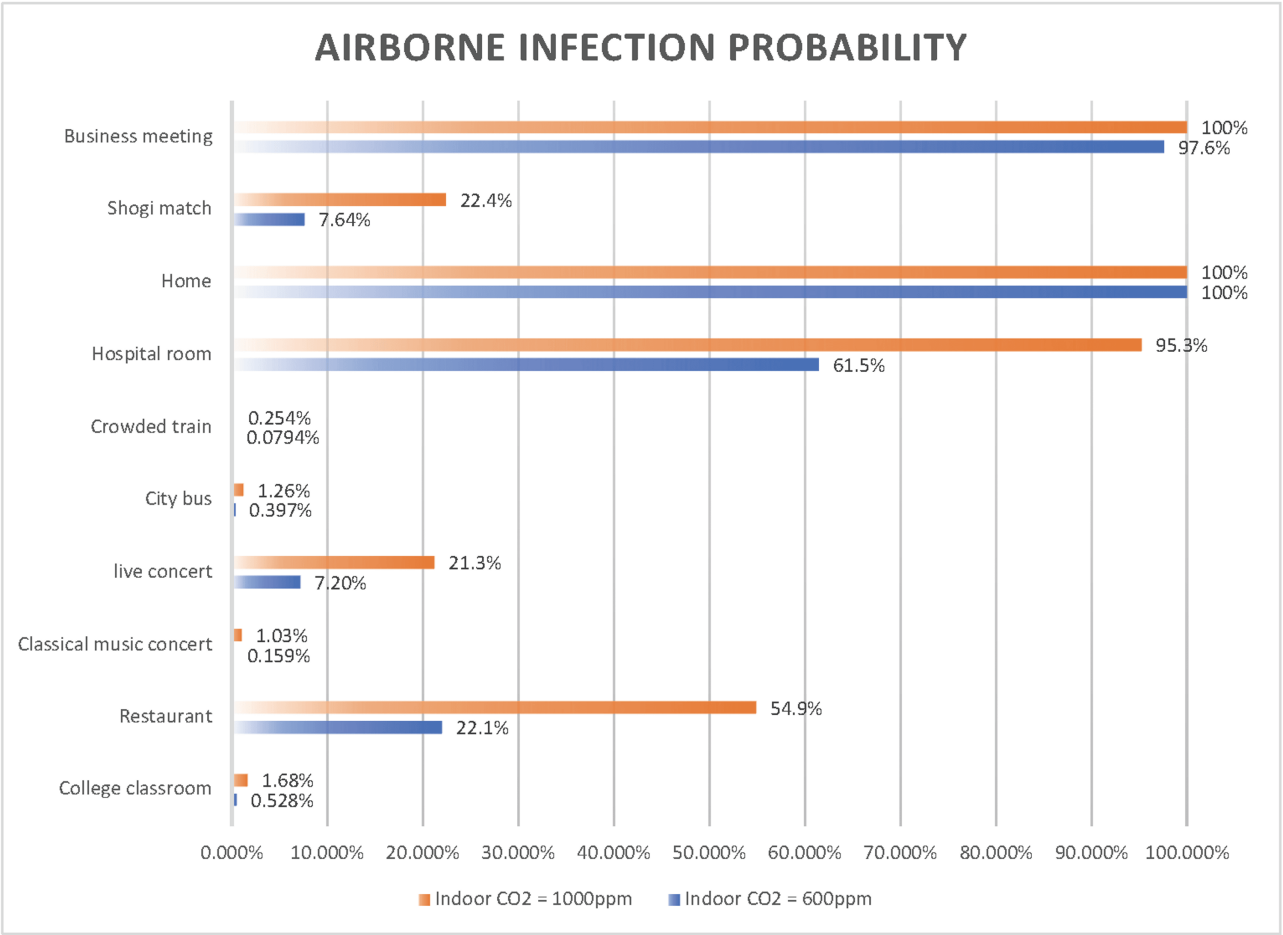
Comparison of the airborne transmission probability under indoor CO2 concentration of 600 ppm and 4,000 ppm

**Figure 16:**
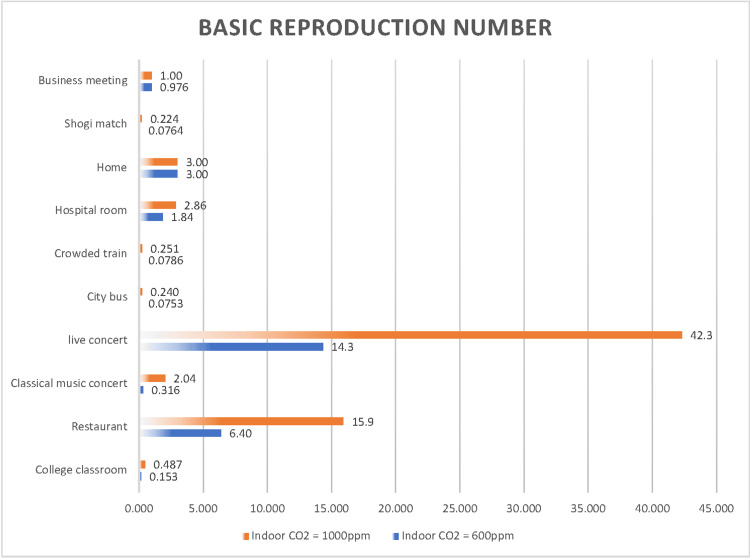
Comparison of the basic reproduction number under indoor CO2 concentration of 600 ppm and 4,000 ppm

We also examined the effects of ventilation in each scenario (Figure 15 and Figure 16), comparing the risk of airborne transmission when neither the infected nor susceptible person wore a mask with the risk when both individuals wore surgical masks.

The probability of airborne transmission was significantly reduced in a hospital room (34%), in a restaurant (33%), during a shogi match (15%), and at a live concert (14%), indicating that surgical masks are highly effective in these settings. In other scenarios, such as in a college classroom (1.2%), at a classical music concert (0.87%), on a city bus (0.87%), on a crowded train (0.17%), at home (0.0%), and in a business meeting (2.4%), improved ventilation alone did not significantly reduce the risk of airborne SARS-CoV-2 transmission. However, this does not imply that improving ventilation is unnecessary in such scenarios. In high-risk scenarios, such as at home or during business meetings, ensuring sufficient ventilation while wearing surgical masks can effectively reduce the infection risk.

This study investigated 10 representative scenarios, with the parameters for each outlined in Table 1. In the 10 scenarios presented, it is conceivable that anyone can readily predict the airborne transmission probability of SARS-CoV-2 using a CO_2_ concentration meter alongside the graphs provided in this study. CO_2_ concentration meters are available on the market with varying degrees of accuracy; however, using a meter employing the nondispersive infrared method for higher precision is recommended. Note that these parameters may vary slightly depending on the circumstances; therefore, to estimate accurate airborne transmission probabilities and R_0_, inserting context-specific parameters into Equation (7) is necessary.

As previously discussed, mask-wearing and improved ventilation can significantly reduce the risk of airborne transmission of SARS-CoV-2. However, the cost-effectiveness of these measures must be carefully evaluated. The disadvantages of mask-wearing include the financial cost of surgical or N95 masks, discomfort associated with CO_2_ accumulation inside the mask, and potential reductions in work efficiency. For ventilation, drawbacks include decreased indoor heating and cooling efficiency, increased electricity costs, elevated CO_2_ emissions, and potential health risks from the introduction of allergens or outdoor air pollutants into the indoor environment. On the other hand, the consequences of SARS-CoV-2 infection include risks of severe illness, deterioration of physical health, medical expenses, and financial losses due to work absences. The relative importance of these trade-offs depends on various factors, including national and regional policies, indoor environmental conditions, the degree of SARS-CoV-2 prevalence, individual vaccination status, underlying health conditions, age, and social responsibilities. Consequently, a comprehensive and contextual assessment is essential for decision-making. 

Limitations

This study has several limitations. The Wells-Riley model presumes that the air within a room is thoroughly mixed to ensure uniform aerosol distribution, making it applicable only to airborne transmission and not to droplet or contact transmission. Consequently, the modified Wells-Riley model incorporating indoor CO_2_ levels should not be used in scenarios where droplet or contact transmission predominates. Furthermore, it assumes steady-state conditions and is unsuitable for areas with significant movement of individuals or outdoor environments. It is also inapplicable in indoor spaces where devices or equipment emit significant amounts of CO_2_, as this can lead to inaccuracies in airborne infection probability calculations.

The model's estimation of airborne infection probability and R_0_ for the SARS-CoV-2 Omicron variant relied on the quantum generation rate (q value) from Dai and Zhao (2023). However, q values for newer SARS-CoV-2 strains like XBB.1.5 and BQ.1.1 are not yet available. Future research incorporating these updated q values may provide clearer insights. Additionally, the model distinguished quantum generation rates based on the presence or absence of conversation. However, these rates vary with factors such as the language spoken, voice volume, and speech frequency. For instance, languages with many plosive or fricative sounds, louder speech, or scenarios involving shouting or singing likely result in higher airborne transmission risks and R_0_ than predicted.

Another critical limitation involves the pulmonary ventilation rate, a key parameter for predicting infection probability. The commonly used rate of 0.48 m^3^/hour is based on an average adult male, potentially underestimating infection risks for populations with lower body weights, such as women or children. Similarly, the CO_2_ emission rate, another parameter in the modified Wells-Riley model, may vary across populations. The model also does not account for reductions in airborne transmission risk due to filtration or ultraviolet disinfection systems.

The estimation of R_0_ using the modified Wells-Riley model assumes homogeneous indoor conditions with uniform viral particle distribution. In reality, airflow dynamics create "hotspots" where viral particles accumulate, especially in areas of higher population density, potentially leading to R_0_ values that exceed model predictions. Environmental factors such as humidity and temperature, which influence viral survival rates, are also not considered in the model, potentially causing discrepancies between predicted and actual R_0_ values.

Finally, host-specific factors such as vaccination status, prior SARS-CoV-2 infection, susceptibility, and overall health can significantly influence infection dynamics. In populations with high vaccination rates or prior infections, the actual airborne transmission risk and R_0_ may be lower than predicted. Conversely, in groups with high proportions of vulnerable individuals (e.g., the elderly, infants, cancer patients, or immunosuppressed individuals in long-term care facilities or hospitals), the transmission risk and R_0_ are likely to exceed predictions.

## Conclusions

Our extensive study employing the modified Wells-Riley equation to determine the airborne transmission risk of SARS-CoV-2 in varied indoor environments has yielded critical insights into infection control strategies. Graphical representations were provided for each scenario to predict the SARS-CoV-2 transmission rates using CO_2_ concentration meters. Under indoor CO_2_ concentrations of 1,000 ppm, the probability of airborne transmission of SARS-CoV-2 was found to be high in business meetings, homes, and hospital rooms; moderate in restaurants, audience-participatory live concerts, and shogi matches; and low in crowded trains, buses, classical music concerts, and college classrooms. Conversely, R_0_ was notably high for audience-participatory live concerts, followed by restaurants, suggesting a greater likelihood of cluster transmissions in these scenarios. We have determined that CO_2_ monitoring when leveraged as an indirect indicator of ventilation adequacy, serves as a valuable tool in mitigating the risk of airborne transmission of the virus. In conclusion, this study emphasizes the need for a comprehensive strategy that includes CO_2_ monitoring, wearing masks, and improving ventilation to reduce the spread of SARS-CoV-2 indoors. It offers practical guidance for identifying high-risk environments and helps policymakers make well-informed decisions to implement effective public health measures.
